# *MECOM* amplified endometrial cancer, a novel subset of copy number high tumors associated with poor prognosis

**DOI:** 10.1016/j.gore.2025.101993

**Published:** 2025-11-16

**Authors:** Shariska P. Harrington, Jacqueline Romani, Aminah Jatoi, S.John Weroha, Andrea Mariani, William A. Cliby, Jamie N. Bakkum-Gamez, Dineo Khabele, Alexandre Gaspar-Maia

**Affiliations:** a Department of Obstetrics and Gynecology, Division of Gynecologic Oncology, Mayo Clinic, Rochester, MN, USA; b Department of Lab Medicine and Pathology, Division of Experimental Pathology, Mayo Clinic, Rochester, MN, USA; c Department of Oncology, Division of Medical Oncology, Mayo Clinic, Rochester, MN, USA; d Department of Obstetrics and Gynecology, Division of Gynecologic Oncology and Siteman Cancer Center, St. Louis, MO, USA

**Keywords:** MECOM, Endometrial cancer, Copy number high, Poor prognosis

## Abstract

**Objective:**

Copy number high (CNH) endometrial cancer (EC) is an aggressive molecular subgroup characterized by TP53 mutations and relative chemoresistance. CNH EC with cyclin E1 gene (*CCNE1*) amplification and erythroblastic oncogene B *(ERBB2)* amplification are associated with poor clinical outcomes. *MECOM*, a complex locus of MDS1 (myelodysplasia syndrome 1) and EVI1 (ecotropic virus integration site 1), has recently been associated with poor prognosis in ovarian cancer. Our objective was to evaluate clinical outcomes of *MECOM*, *CCNE1* and *ERBB2* amplified ECs and to provide a literature review on the role of *MECOM* in gynecologic cancers.

**Methods:**

Copy number variation and molecular subtype classification were extracted from The Cancer Genome Atlas for 529 ECs. Amplification status was determined for *MECOM*, *CCNE1* and *ERBB2.* Measured clinical outcomes were overall and progression-free survival, covariates included race, stage at diagnosis, and tumor histology. A comprehensive search of peer-reviewed articles was undertaken to summarize evidence on the role of *MECOM* in gynecologic cancers.

**Results:**

Of all ECs profiled, *MECOM* was the most frequently amplified gene. Notably, 35% of CNH ECs were *MECOM* amplified. In multivariate analysis, *MECOM* amplification without co-amplification of *CCNE1* or *ERBB2* was associated with an increased risk of death and recurrence, HR 2.3 [1.17–4.62], p = 0.0163 and HR 2.07 [1.08–3.98], p = 0.0282, respectively. The literature review identified 19 relevant studies with inconsistent evidence on *MECOM’s* role in carcinogenesis.

**Conclusions:**

ECs with *MECOM* amplification are associated with poor clinical outcomes, even in the absence of *CCNE1* or *ERBB2* amplification. The current literature is limited, and further studies are warranted to determine the role of *MECOM* amplification in ECs.

## Highlights

• *MECOM* is the most frequently amplified gene in endometrial cancers (EC) of the TCGA.
• 35 % of copy number high endometrial cancers have amplification in *MECOM*.
• *MECOM* amplified EC is associated with a two-fold increase risk in death and recurrence.
• Co-amplification of *CCNE1* and *ERBB2* occur in ECs with *MECOM* amplification.

## Graphical abstract

**Figure:**
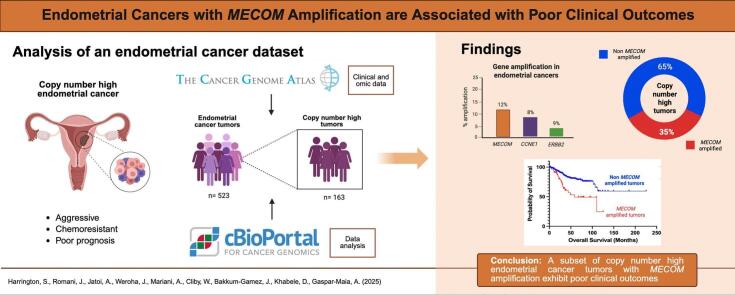

## 1 Introduction

Endometrial cancer (EC) mortality rates are increasing by 1.8 % annually and in 2024, endometrial cancer surpassed ovarian cancer as the leading cause of death from gynecologic malignancy (Siegel et al., 2024). One factor contributing to the rising EC mortality rate, is an increasing incidence of copy number high (CNH) EC. This is because patients with CNH endometrial cancer are more likely to have advanced stage at diagnosis and are less likely to respond to platinum-based chemotherapy, two factors that contribute to high rates of relapse and death (Tortorella, 2018, Bakkum-Gamez, 2014). This lack of highly effective treatment strategies for advanced stage or recurrent CNH endometrial cancer represents a significant clinical problem, therefore, efforts to identify drivers and molecular targets of CNH EC are needed.

A subset of CNH endometrial cancer characterized by amplification in *ERBB2* (commonly referred to as *HER2* gene), is associated with poor outcomes (Santin, 2005). Combination therapy with HER2-directed monoclonal antibodies and standard platinum and taxane-based chemotherapy has significantly improved treatment outcomes in advanced and recurrent *HER2* amplified CNH endometrial cancer (Fader, 2020). Another subset of CNH endometrial cancer characterized by amplification in *CCNE1* is also implicated in poor prognostic CNH EC (Nakayama, 2016). However, strategies for targeting *CCNE1* amplified tumors remain elusive. Thus, efforts to identify additional subsets of CNH EC with therapeutic potential are warranted to improve treatment outcomes.

*MECOM,* first identified as a marker of poor prognosis in leukemia, is a complex locus resulting from the fusion of two genes: *MDS1* (myelodysplasia syndrome 1) and *EVI1* (ecotropic virus integration site 1), located on chromosome 3 (3q26.2) (Soderholm, 1997). *MECOM* has recently been classified as a master transcription factor and an oncogene of interest in high grade serous ovarian cancer (Bleu, 2021). Studies have implicated *MECOM* and/or *EVI1* overexpression in increased cell proliferation, migration and reduced apoptosis, supporting *MECOM*’s role in ovarian cancer tumorigenesis (Bleu, 2021, Brooks, 1996, Chen, 2024, Lan, 2024, Ma, 2019, Nameki, 2023, Rockfield et al., 2019, Wang, 2021, Bard-Chapeau, 2012). Successfully targeting *MECOM* and/or *EVI1* expression with epigenetic drugs suggests a role for MECOM directed therapy in high grade serous ovarian cancer (Chen, 2024, Nameki, 2023). However, there is a paucity of data of the role of *MECOM* in CNH EC. The objective of this study was to characterize a novel subset of CNH endometrial cancer, *MECOM* amplified, and to examine the overlap of *MECOM* amplification with amplification in *CCNE1* and *ERBB2* using a publicly available dataset. Given the emerging role of *MECOM* in gynecologic malignancies, we also seek to provide a review of the current literature on the role of *MECOM* amplification in gynecologic malignancies.

## 2 Methods

### 2.1 TCGA data

This is a retrospective, secondary analysis of a publicly available dataset. Published copy number, demographic and clinical data were extracted from The Cancer Genome Atlas (TCGA) PanCancer Atlas dataset of 529 endometrial cancers diagnosed between 1995 and 2011 (Cancer Genome Atlas Research, 2013). Inclusion criteria for tumors in the PanCancer Atlas were based on the broader TCGA project rules to ensure high-quality, clinically annotated tumor samples including a confirmed diagnosis, treatment naïve tissue and availability of clinical data. We used the PanCancer Atlas as it includes a diverse cohort of patients with complete copy number variation data. Amplifications in *MECOM*, *CCNE1* and *ERBB2* were determined using Genomic Identification of Significant Targets in Cancer (GISTIC) 2.0 prediction by TCGA as previously described (Mermel, 2011). A value of ‘2′ estimated by GISTIC 2.0 was considered high level amplification, as previously established (Cancer Genome Atlas Research, 2013, Beroukhim, 2007, Cancer Genome Atlas Research, 2008). All other values were classified as non-amplified. Oncoprints were generated to demonstrate overlapping of *MECOM*; *CCNE1, and ERBB2* gene amplification in endometrial cancers. To substantiate gene amplification, mRNA expression levels for *MECOM*, *CCNE1, and ERBB2* amplified tumors vs non-amplified tumors were obtained using RNA-Seq by Expectation-Maximization (batch normalized from Illumina HiSeq_RNASeqV2). *MECOM* amplification was then stratified by stage at diagnosis, tumor histology, molecular subtype, and self-reported participant race. Ethnicity was not included in the analyses because it was not reliably collected in this dataset (28.7 % not reported). To corroborate differences found by self-reported race, self-reported race was correlated to genetic ancestry. Genetic ancestry as reported in the PanCancer Atlas was determined by principal component analysis of germline SNPs from matched normal DNA, using reference populations from the 1000 Genomes Project, as previously described (Carrot-Zhang, 2020).

Statistical analysis: Demographic and clinical data were stratified by *MECOM* amplified vs non-amplified and tested via chi-squared analysis. *MECOM, CCNE1* and *ERBB2* amplification status, dichotomized by amplified vs non-amplified, was used to generate overall survival (OS) and progression-free survival (PFS) curves by Kaplan-Meier analysis and differences between curves were determined by log-rank tests. Race, histology, and stage (early—stage I/II, advanced—stage III/IV) were included as covariables in Cox proportional hazards regression models to calculate Hazard Ratios (HR) and corresponding 95 % confidence intervals (CI). Co-variates were selected based on univariate analysis of demographic and clinical variables with p-value < 0.20. Data analysis was completed using SAS software. A threshold of p < 0.05 was used to indicate statistical significance.

### 2.2 Literature review

A comprehensive search of PubMed (MEDLINE) and Ovid Embase was conducted in December 2024. The search strategy was designed and conducted by an experienced biomedical librarian with input from the study’s principal investigator (SPH). Controlled vocabulary supplemented with keywords was used to search for studies describing *MECOM* gene expression and/or amplification in endometrial, ovarian, and fallopian tube cancers. Ovarian and fallopian tube cancers were included in this literature search due to molecular similarities between high grade serous ovarian cancer and CNH endometrial cancer. The actual strategies listing all search terms used and how they are combined are available in the supplementary appendix (S1).

This search yielded 18 abstracts from PubMed and 35 from Ovid Embase. Duplicate abstracts were excluded, and a total of 44 abstracts were reviewed for relevance to *MECOM* amplification and gene expression in ovarian, fallopian tube and endometrial cancers. Ten conference abstracts were excluded due to absence of corresponding peer-reviewed publications. Twelve articles were excluded after review revealed a focus on non-gynecological malignancies with cursory mention of ovarian, fallopian tube or endometrial cancer and three review articles were excluded due to absence of original research. A total of 19 original articles were reviewed for relevance of *MECOM* and/or *EV1/MDS1* in tumorigenesis of ovarian, fallopian tube or endometrial cancer. Articles were summarized by cancer type, study design (clinical vs. preclinical), study objective and key findings relevant to gynecologic oncology.

## 3 Results

### 3.1 *MECOM* amplification is associated with CNH endometrial cancer

Demographics and clinical data by *MECOM* amplification status of the 529 patients diagnosed with endometrial cancer included in the TCGA PanCancer Atlas are summarized in Table 1.

**Table 1:** Demographics and Clinical Data by MECOM amplification status.

	Total N = 529	MECOM amplified N = 62	non-MECOM amplified N = 467	P-value
Age at diagnosis# <70 years ≥70 years	367 (70.2 %) 156 (29.8 %)	40 (10.9 %) 22 (14.1 %)	327 (89.1 %) 134 (85.9 %)	0.2998
Stage# Early (I/II) Late (III/IV)	375 (71.7 %) 148 (28.3 %)	32 (8.5 %) 30 (20.3 %)	343 (91.5 %) 118 (79.7 %)	0.0002
Race# White Black Asian American Indian or Alaskan Native Hawaiian or Other Declined	353 (67.5 %) 105 (20.1 %) 20 (3.8 %) 4 (0.8 %) 9 (1.7 %) 32 (6.1 %)	31 (8.8 %) 22 (20.9 %) 0 0 2 (22.2 %) 7 (21.9 %)	322 (91.2 %) 83 (79 %) 20 (100 %) 4 (100 %) 7 (77.8 %) 25 (78.1 %)	0.0022
Tumor histology# Endometrioid Serous Mixed	394 (75.3 %) 108 (20.7 %) 21 (4 %)	23 (5.8 %) 36 (33.3 %) 3 (14.3 %)	371 (94.2 %) 72 (66.7 %) 18 (85.7 %)	<0.0001
Histologic grade^$^ G1 G2 G3	95 (18.6 %) 117 (22.9 %) 300 (58.6 %)	0 3 (2.6 %) 57 (19 %)	95 (100 %) 114 (97.4 %) 243 (81 %)	<0.0001
Molecular Classification^+^ POLEm MSI CNL CNH	49 (9.7 %) 148 (19.1 %) 147 (28.9 %) 163 (32.2 %)	2 (4.1 %) 1 (0.7 %) 1 (0.7 %) 57 (35 %)	47 (95.9 %) 147 (99.3 %) 146 (99.3 %) 106 (65 %)	<0.0001
Additional Treatment*^+^ Yes No	219 (43.8 %) 281 (56.2 %)	28 (12.8 %) 28 (10 %)	191 (87.2 %) 253 (90 %)	0.3210

# missing data for n = 6; ^$^missing data for n = 17; ^+^missing data for n = 22; *includes all treatment received.

The average age at diagnosis was 63.8 years, 67.5 % of patients identified as White, 20.1 % as Black, 3.8% as Asian, 2.5 % as American Indian or Native Hawaiian. Self-reported race was well correlated with genetic ancestry, 96 % of self-reported White participants were found to have European ancestry and 96% of self-reported Black participants had African or African admixed ancestry (S2). Most tumors (75.3%) were endometrioid, 20.7 % serous and 4 % mixed histology. Molecular classification was determined for 96.9 % of tumors; 9.7 % had *POLE* hypermutation (*POLE*m), 19.1 % had microsatellite instability (MSI), 28.9 % were copy number low (CNL) and 32.2 % were copy number high (CNH). Additionally, 71.7 % of patients were diagnosed at early stage (I/II) and 28.3 % at an advanced stage (III/IV).

**Fig. 1:**
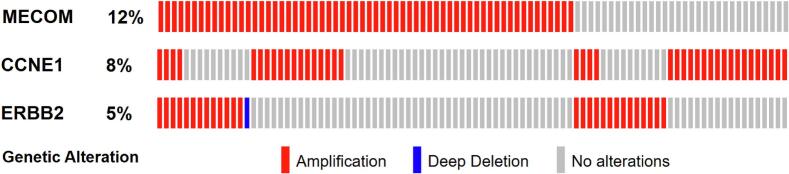
Rates of *MECOM*, *CCNE1*, and *ERBB2* amplification in endometrial cancers of the TCGA. Oncoprint highlighting patients with at least one alteration in the genes: *CCNE1* was concurrently amplified in 29% (18/62) of *MECOM* amplified tumors. *ERBB2* was concurrently amplified in 21% (13/62) of the *MECOM* amplified tumors.

Copy number alteration data was available for 99 % (523/529) of tumors in this cohort. *MECOM* was identified to be the most amplified gene in the entire cohort with 11.9 % (62/523) of tumors exhibiting amplification. (Fig. 1). Co-amplification of *CCNE1* occurred in 29 % (18/62) of *MECOM* amplified tumors and co-amplification of *ERBB2* occurred in 21 % (13/62) of *MECOM* amplified tumors. Overlap of *CCNE1*, *ERBB2* and *MECOM* amplification was seen in only 4 (0.8 %) of all profiled tumors (Fig. 1). *MECOM* amplified tumors were confirmed to have higher MECOM mRNA expression levels vs non-amplified tumors, (5081.4 vs 3806.1 transcripts per million, p = 0.0006) consistent with overexpression. *CCNE1* amplified tumors and *ERBB2* amplified tumors were also confirmed to have higher mRNA expression levels vs non-amplified tumors, 2259.6 vs 544.2 transcripts per million, p < 0.0001 and 75800.2 vs 5371 transcripts per million, p < 0.0001, respectively, consistent with overexpression. When stratified by molecular subtype, race, stage and histology, *MECOM* amplification was most likely to occur in CNH endometrial cancer, tumors obtained from Black women, those diagnosed at advanced stages (III/IV) and in serous tumors. Notably, 93.4 % (57/61) of *MECOM* amplified tumors were CNH tumors and 35 % (57/163) of CNH tumors exhibited *MECOM* amplification. Importantly, endometrial cancers from Black patients were more likely to have amplification in *MECOM* than tumors from White patients, 20.9 % vs 8.8 % respectively, p = 0.0006.

**Fig. 2:**
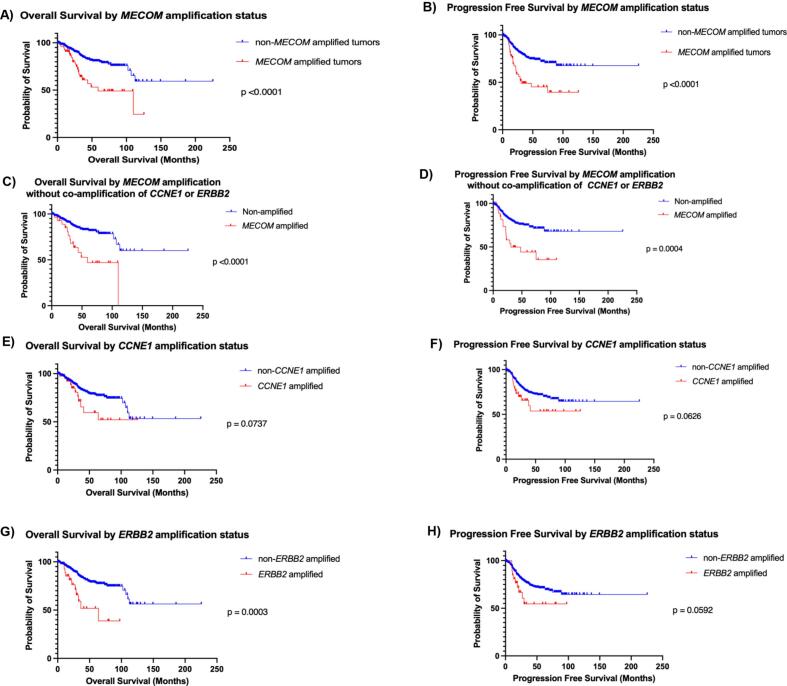
Overall and progression-free survival by amplification status. A) Overall Survival by *MECOM* amplification status for the entire cohort. B) Progression-Free Survival by *MECOM* amplification status for the entire cohort. C) Overall Survival by *MECOM* amplification status for subset of tumors without co-amplification of *CCNE1* or *ERBB2*. D) Progression-Free Survival by *MECOM* amplification for subset of tumors without co-amplification of *CCNE1* or *ERBB2*. E) Overall Survival by *CCNE1* amplification status for the entire cohort. F) Progression-Free Survival by *CCNE1* amplification status for the entire cohort. G) Overall Survival by *ERBB2* amplification status for the entire cohort. H) Progression-Free Survival by *ERBB2* amplification status for the entire cohort.

*MECOM* amplification was associated with worse overall and progression-free survival. The 5-year overall survival in patients with *MECOM* amplified tumors was 49 % vs 81 % in *MECOM* non-amplified tumors p < 0.0001 (Fig. 2A). The 3-year progression-free survival in patients with *MECOM* amplified tumors was 49 % vs 79 % in *MECOM* non-amplified tumors p < 0.0001 (Fig. 2B). After controlling for high-grade histology, Black race and advanced stage at diagnosis, *MECOM* amplification was associated with an increased risk of death HR 2.0 [1.16–3.52], p = 0.0133 and an increased risk of recurrence, HR 2.1 [1.27–3.47], p = 0.0036 (Table 2). In univariate analysis, both advanced stage and high-grade histology are independently associated with an increased risk of death and recurrence (S3). After inclusion of *MECOM* amplification into the cox proportional hazards model, the adjusted hazard ratios for stage and high-grade histology were modestly attenuated, suggesting *MECOM* amplification accounts for a portion of the adverse prognostic effect attributed to stage and high-grade histology (S3).

**Table 2:** Cox Proportional Hazards Models for Progression-Free and Overall Survival by amplification status.

Variable	Unadjusted Hazard Ratio (95 % CI)	p-value	Adjusted* Hazard Ratio (95 % CI)	p-value
Progression-Free Survival				
MECOM amplification *MECOM* amplification# *CCNE1* amplification *ERBB2* amplification	2.59 [1.68–3.98]	<0.0001	2.10 [1.27–3.47]	0.0036
	2.62 [1.51–4.55]	0.0006	2.07 [1.08–3.98]	0.0282
	1.69 [0.97–2.96]	0.0657	0.96 [0.51–1.89]	0.9408
	1.85 [0.97–3.54]	0.0632	1.07 [0.51–2.22]	0.8624
Overall Survival				
MECOM amplification *MECOM* amplification# *CCNE1* amplification *ERBB2* amplification	2.66 [1.62–4.36]	<0.0001	2.02 [1.16–3.52]	0.0133
	3.08 [1.68–5.65]	0.0003	2.32 [1.17–4.62]	0.0163
	1.77 [0.94–3.34]	0.0777	0.84 [0.39–1.78]	0.6411
	3.06 [1.62–5.77]	0.0006	1.47 [0.70–3.09]	0.3152

Multivariate models adjusting for high-grade histology, Black race and advanced stage at diagnosis.

# MECOM amplification without co-amplification of CCNE1 or ERBB2.

Notably, 56 % (35/62) of *MECOM* amplified tumors did not harbor amplification in either *CCNE1* or *ERBB2.* The 5-year survival in patients with tumors that only had *MECOM* amplification was 63 % vs 86 % in patients without amplification in *CCNE1*, *ERBB2* or *MECOM,* p < 0.0001 (Fig. 2C). The 3-year progression-free survival in patients with *MECOM* amplified tumors was 50 % vs 79 % in patients without amplification in *CCNE1*, *ERBB2* or *MECOM,* p = 0.0004 (Fig. 2D). After controlling for high-grade histology, Black race and late stage at diagnosis, *MECOM* amplification without co-amplification of either *CCNE1* or *ERBB2* was associated with an increased risk of death HR 2.3 [1.17–4.62], p = 0.0163 and an increased risk of recurrence HR 2.07 [1.08–3.98], p = 0.0282 (Table 2).

*CCNE1* was amplified in 7.6 % (40/523) of the tumors in the cohort, of which 97.5 % (39/40) were found in CNH endometrial cancer. The 5-year overall survival in patients with *CCNE1* amplified tumors was 65 % vs 82 % in *CCNE1* non-amplified tumors, p = 0.0737 (Fig. 2E). The 3-year progression-free survival in patients with *CCNE1* amplified tumors was 66 % vs 76 % in *CCNE1* non-amplified tumors, p = 0.0626 (Fig. 2F). After controlling for high-grade histology, Black race, and late stage at diagnosis, *CCNE1* amplification was not associated with increased risk of death HR 0.84 [0.39–1.78], p = 0.6411 or recurrence HR 0.96 [0.51–1.89], p = 0.9408 (Table 2). However, the estimated post hoc power to detect an overall survival difference in *CCNE1* amplified tumors was approximately 43 %, indicating a low sensitivity to detect a survival difference likely due to sample size.

*ERBB2* was amplified in 5.2 % (27/523) of tumors in the cohort, and in the subset of *ERBB2* amplified tumors, 92.6 % (25/26) were found in CNH endometrial cancer. The 5-year overall survival in patients with *ERBB2* amplified tumors was 52 % vs 83 % in *ERBB2* non-amplified tumors, p = 0.0003 (Fig. 2G). The 3-year progression-free survival in patients with *ERBB2* amplified tumors was 54 % vs 76 % in *ERBB2* non-amplified tumors, p = 0.0592 (Fig. 2H). Notably, after controlling for high-grade histology, Black race, and late stage at diagnosis, *ERBB2* amplification was not associated with an increased risk of death HR 1.47 [0.70–3.09], p = 0.3152 or recurrence HR 1.07 [0.51–2.22], p = 0.8624 (Table 2).

### 3.2 Studies of *MECOM* in gynecologic cancer are limited

To determine the role of *MECOM* and/or *EV1/MDS1* in endometrial cancer, we performed a broad literature review inclusive of the most common gynecologic cancers (endometrial/uterine and ovarian/fallopian tube). A total of 19 original articles were reviewed and summarized in Table 3.

**Table 3:** Literature review of *MECOM* in gynecologic cancers.

Article	Cancer type	Study design	Objective	Key findings
Bard-Chapeau et al. (2012)	Epithelial ovarian cancer	Preclinical	Investigate the interaction between EVI1 and FOS protein.	EVI1 and FOS protein have synergistic interaction. *EVI1* regulates cell adhesion, proliferation and colony formation in epithelial ovarian cancer.
Bleu, M et al (2021)	Epithelial ovarian cancer	Preclinical	Investigate the interaction between *PAX8* and *MECOM*.	*MECOM* and *PAX8* sustain in vivo tumor growth. High *PAX8/MECOM* expression identifies subset of poor prognosis in TCGA.
Brooks, D. J. et al (1996)	Epithelial ovarian cancer	Preclinical	Investigate expression of EVI1 in tumor vs. normal tissue.	Overexpression of EVI1 in comparison to normal tissues suggests a role for EVI1 in solid tumor carcinogenesis or progression.
Chen, Y. et al (2024)	Epithelial ovarian cancer	Preclinical	Investigate the effect of epigenetic drugs on MECOM expression.	Inhibition of MECOM expression with Bromodomain and extraterminal (BET) inhibitor JQ-1 slows ovarian cancer progression.
Dutta, P. et al (2013)	Epithelial ovarian cancer	Preclinical	Characterize the role of EVI1 variants.	Alternative splicing of EVI1 has differential function and is context dependent.
Jank, P. et al (2023)	Epithelial ovarian cancer	Clinical	Correlate expression of EVI1 and PARP1 to clinical outcomes.	High EVI1 and PARP1 protein expression identifies a subgroup of patients with good prognosis.
Jazaeri, A. et al. (2010)	Epithelial ovarian cancer	Preclinical	Investigate the functional role of two major isoforms of EVI1.	Knockdown of EVI1 isoforms had no effect on proliferation, apoptosis or markers of DNA damage.
Lan, N. et al (2024)	Epithelial ovarian cancer	Clinical	Correlate MECOM isoform expression to clinical outcomes.	Elevated expression of certain MECOM isoform transcripts correlated with poor survival in ovarian cancer.
Lu, Y. et al (2024)	Endometrial cancer	Clinical	Correlate MECOM expression to clinical outcomes.	High MECOM expression in uterine carcinosarcoma (UCS) is associated with shorter disease-free survival than low MECOM expression in UCS.
Lou, M. et al (2024)	Endometrial cancer	Preclinical	Investigate the functional role of *MECOM*.	*MECOM* knockout studies demonstrated reduced proliferation, migration and increased apoptosis. *MECOM* plays a key role in the development and progression of endometrial cancer.
Ma, H. et al (2019)	Epithelial ovarian cancer	Preclinical	Characterize interaction between PDZ binding kinase (*PBK*) and *EVI1*.	*EVI1* directly binds to the promoter region of *PBK* and promotes transcription. High co-expression of *PBK* and *EVI1* are seen in patients with poor prognosis.
Morishita, K. et al (1990)	Endometrial cancer	Preclinical	Investigate expression of EVI1 in EC cell lines.	EVI1 is expressed at low levels in HEC-1-A cells and at high levels in HEC-1-B cells.
Nameki, R. et al (2023)	Epithelial ovarian cancer	Preclinical	Investigate the effect of epigenetic drugs on MECOM expression.	Treatment with CDK7 inhibitor (THZ1) resulted in decreased cell survival and decreased expression of MECOM in ovarian cancer cell lines but no effect in benign fallopian tube cell lines.
Nanjundan, M. et al (2007)	Epithelial ovarian cancer	Clinical	Correlate *EVI1* gene copy number and *MDS1/EVI1* to clinical outcomes.	Elevated *MDS1/EVI1* transcript levels are associated with increased survival duration.
Rockfield, S. et al (2019)	Fallopian tube secretory epithelial cells	Preclinical	Role of upregulation of *EVI1* on migration of fallopian tube secretory epithelial cells.	Upregulation of *EVI1* elicited elevated migratory capacity and increased migration in fallopian tube secretory epithelial cells.
Sayadi, A. et al (2016)	Epithelial ovarian cancer	Preclinical	Investigate the functional role of *MECOM* isoforms.	Two major isoforms of the *MECOM* gene, EVI1 and EVI1Δ324, share a common C-terminus ZNF, however, EVI1 has as a specific N-terminus ZNF domain associated with high recombination rates and potential for differences in oncogenicity.
Wang, Z et al. (2019)	Epithelial ovarian cancer	Preclinical	Investigate the interaction between *EVI1* and estrogen.	Knockdown of *EVI1* decreases tumor progression. Estrogen rescues tumor proliferation. *EVI1* expression is correlated with estrogen receptor expression. *EVI1* functions as a novel regulator of the estrogen signaling network in ovarian cancer.
Weberpals, J. et al (2021)	Epithelial ovarian cancer	Clinical	Correlate *MECOM* amplification to clinical outcomes.	*MECOM* amplification is more likely to occur poor (< 6 months) vs. good (≥12 months) chemotherapy responders.
Yin, F. et al (2018)	Epithelial ovarian cancer	Clinical .	Correlate MECOM expression to clinical outcomes.	High MECOM mRNA levels were independently associated with improved disease-free survival.

## 4 Ovarian cancer

In summary, most preclinical studies support the role of *MECOM* and/or *EVI1* overexpression in ovarian cancer tumorigenesis. Several clinical studies report an association of *MECOM* and/or *EVI1* overexpression to poor clinical outcomes (Bleu, 2021, Brooks, 1996, Chen, 2024, Lan, 2024, Ma, 2019, Nameki, 2023, Rockfield et al., 2019, Wang, 2021, Bard-Chapeau, 2012). However, in contrast, a few clinical studies report favorable clinical outcomes with *MECOM* and/or *EVI1* overexpression (Jank, 2023, Yin, 2019, Nanjundan, 2007, Jazaeri, 2010). Most notably, this literature review has highlighted the existence of three isoforms, *MDS1/EVI1*, *EVI1* and EVI1Δ324 arising from alternative splicing of the *MDS1* and *EVI1* complex locus. (Lan, 2024, Dutta, 2013, Sayadi, 2016, Nameki, 2021).The function of these isoforms are incompletely characterized in the literature and may be context dependent. Thus, the mixed literature review findings on the role of *MECOM* and/or *EVI1* overexpression in ovarian tumorigenesis and its association with clinical outcomes, are likely due in part to a lack of identification of which specific isoform was investigated (Nameki, 2021).

## 5 Endometrial cancer

Only three articles were focused on the role of *MECOM* in endometrial cancer; one clinical study and two studies focused on preclinical analysis. An analysis of TGCA data determined that high *MECOM* expression in uterine carcinosarcoma was associated with shorter disease-free survival than low *MECOM* expression (Lu, 2024). Another study investigated *EVI1* expression in endometrial cancer cell lines. HEC-1-A and HEC-1-B are human endometrial adenocarcinoma cell lines originally derived from the same patient with a stage IA, grade 2 endometrial carcinoma. This study revealed that *EVI1* is expressed in low levels in HEC-1-A cells and at high levels in HEC-1-B cells, however, the basis for differential expression was not determined (Morishita, 1990). In the third study, Lou et al demonstrated that *MECOM* knockout in endometrial cancer cell lines resulted in reduced cell proliferation, migration and increased apoptosis; while *MECOM* overexpression resulted in the opposite, validating *MECOM*’s role in endometrial cancer tumorigenesis (Lou, 2024).

## 6 Discussion

Here, we propose that *MECOM* amplified endometrial cancer is a novel subset of CNH tumors. Based on the TCGA PanCancer Atlas, patients with *MECOM* amplified tumors had a two-fold increased risk of death and recurrence in multivariate analyses. Notably, *MECOM* amplification co-occurred with *CCNE1* and *ERBB2*, two subsets of endometrial cancer known for their poor prognosis in CNH tumors. Furthermore, even in the absence of co-amplification of *CCNE1* or *ERBB2*, *MECOM* amplification alone conferred a similar two-fold increase in risk, suggesting that *MECOM* amplification may independently identify an aggressive subset of endometrial cancers.

Although prior studies have reported that *CCNE1* amplification is associated with poor prognosis in endometrial cancer, we did not observe an increased risk of death or recurrence in this cohort. This discrepancy may reflect limited statistical power to detect modest prognostic differences, as suggested by our post hoc analysis. Similarly, *ERBB2* amplification was not associated with poor prognosis in this study. While univariate analysis suggested an approximately threefold increased risk of death, this association was not maintained after multivariable adjustment. These findings align with recent reports, including analyses of 684 endometrial carcinomas from TCGA and tumors from patients enrolled in the PORTEC-3 trial, which demonstrated that HER2 status lacks independent prognostic value after accounting for established clinicopathologic and molecular factors (Vermij, 2020). Likewise, a large study of 806 endometrial cancers found no independent association between HER2 status and prognosis in multivariable analysis *PMID 39374474*. Although these investigations evaluated HER2 protein expression, HER2 positivity was corroborated by *ERBB2* amplification using dual in situ hybridization.

The functional role of *MECOM* and/or *EVI1* expression in preclinical studies, specifically in endometrial cancer, is limited. To our knowledge, there are no reported preclinical studies investigating targeted therapy of *MECOM* amplification in endometrial cancer. However, in ovarian cancer cell lines, treatment with CDK7 inhibitor (THZ1) resulted in decreased expression of MECOM and decreased cell survival with no off-target effect in benign fallopian tube cell lines (Nameki, 2023). Similarly, JQ-1, a bromodomain and extraterminal (BET) inhibitor has been shown to inhibit the expression of MECOM and ovarian cancer tumorigenesis (Chen, 2024). This suggests a potential role for transcriptional targeted inhibitors in the treatment of *MECOM* amplified endometrial cancers.

This study has several limitations. It relies on TCGA data, which, although comprehensive, may not fully represent the clinical and molecular heterogeneity of endometrial cancer in the general population. In addition, detailed treatment, recurrence, and follow-up data are limited, restricting the ability to evaluate how *MECOM* or other gene amplifications influence therapeutic response or long-term outcomes. Finally, the relatively small number of cases with amplifications may limit statistical power to detect prognostic associations. Despite these limitations, this study leverages a large, well-characterized cohort with comprehensive genomic profiling, enabling detailed evaluation of CNH endometrial cancers and *MECOM*, *CCNE1*, and *ERBB2* gene amplifications. The availability of integrated clinical, molecular, and outcome data allows for multivariable analyses that account for established prognostic factors, strengthening the robustness and interpretability of our findings. Additionally, the comprehensive literature search was completed with the assistance of an experienced biomedical librarian, ensuring all relevant articles to our knowledge were included and revealed the novelty of the role of *MECOM* in endometrial cancer. Future directions include investigating clinical outcomes of specific isoforms in CNH endometrial cancer.

We recognize that while preclinical studies have shown that *MECOM* amplified tumors are sensitive to transcriptional targeted inhibitors in high grade serous ovarian cancer, there is a paucity of data in preclinical models of endometrial cancer. More research is needed to determine the feasibility of therapeutically targeting MECOM in endometrial cancer. Rates of co-amplification of *ERBB2* and *MECOM* may represent a subgroup of patients who might benefit from MECOM directed therapy after they have progressed on HER2-targeted therapy. Similarly, patients with co-amplification in *CCNE1* and *MECOM* may benefit also from MECOM directed therapy, as strategies for targeting *CCNE1* amplified tumors remain elusive. We postulate that investigating vulnerabilities of *MECOM* amplified tumors will be a useful strategy to reduce mortality associated with CNH endometrial cancer. As such, this study is intended to be hypothesis generating and encourage further studies.

## 7 Conclusion

Our study proposes *MECOM* amplified endometrial cancer as a novel subset of CNH tumors with poor prognosis. The high rate of *MECOM* amplified tumors (35 %) in CNH EC suggests that these tumors may harbor a unique, unidentified vulnerability with therapeutic potential. Further studies are warranted to explore the role of *MECOM* and its isoforms in CNH endometrial cancer.

## CRediT authorship contribution statement

**Shariska P. Harrington:** Writing – review & editing, Writing – original draft, Visualization, Validation, Software, Methodology, Investigation, Funding acquisition, Formal analysis, Conceptualization. **Jacqueline Romani:** Writing – review & editing. **Aminah Jatoi:** Writing – review & editing, Supervision. **S.John Weroha:** Writing – review & editing, Supervision, Methodology. **Andrea Mariani:** Writing – review & editing. **William A. Cliby:** Writing – review & editing, Supervision. **Jamie N. Bakkum-Gamez:** Writing – review & editing, Supervision. **Dineo Khabele:** Writing – review & editing, Methodology. **Alexandre Gaspar-Maia:** Writing – review & editing, Methodology.

## Funding

SPH is supported by the Paul Calabresi Program in Clinical/Translational Research at the 10.13039/100000871Mayo Clinic Comprehensive Cancer Center [K12CA090628].

## Declaration of competing interest

The authors declare that they have no known competing financial interests or personal relationships that could have appeared to influence the work reported in this paper.
