# Supplementary material for: MECOM amplified endometrial cancer, a novel subset of copy number high tumors associated with poor prognosis
Source: Gynecol Oncol Rep. 2025 Nov 16;62:101993. doi: 10.1016/j.gore.2025.101993 (PMC12670447; doi:10.1016/j.gore.2025.101993)
Supplement: Supplementary Data 1 [file mmc1.docx]

Supplementary Appendix 1. MECOM literature search strategies

PubMed:

("MDS1 and EVI1 Complex Locus Protein"[Mesh] OR "MECOM protein, human" [Supplementary Concept] OR MECOM [tiab]) AND ("Gene Expression"[Mesh] OR expression [tiab]) AND ("Endometrial Neoplasms"[Mesh] OR "Ovarian Neoplasms"[Mesh] OR "Genital Neoplasms, Female"[Mesh] OR endometri* [tiab] OR ovar* [tiab] OR fallopian [tiab] OR vagin* [tiab] OR uter* [tiab])

English and Human = 18

Embase <1974 to 2024 December 17>

1          exp "MDS1 and EVI1 complex locus protein"/ or MECOM.ti,ab.       883

2          exp gene expression/ or expression.ti,ab.      4133673

3          exp endometrium cancer/      72293

4          exp ovary cancer/       163025

5          exp female genital tract cancer/ or endometri*.ti,ab. or ovar*.ti,ab. or fallopian.ti,ab. or vagin*.ti,ab. or uter*.ti,ab.       1063099

6          3 or 4 or 5       1063099

7          1 and 2 and 6  41

8          limit 7 to (human and english language)       35
