# Supplementary figures and images for: MECOM amplified endometrial cancer, a novel subset of copy number high tumors associated with poor prognosis
Source: Gynecol Oncol Rep. 2025 Nov 16;62:101993. doi: 10.1016/j.gore.2025.101993 (PMC12670447; doi:10.1016/j.gore.2025.101993)

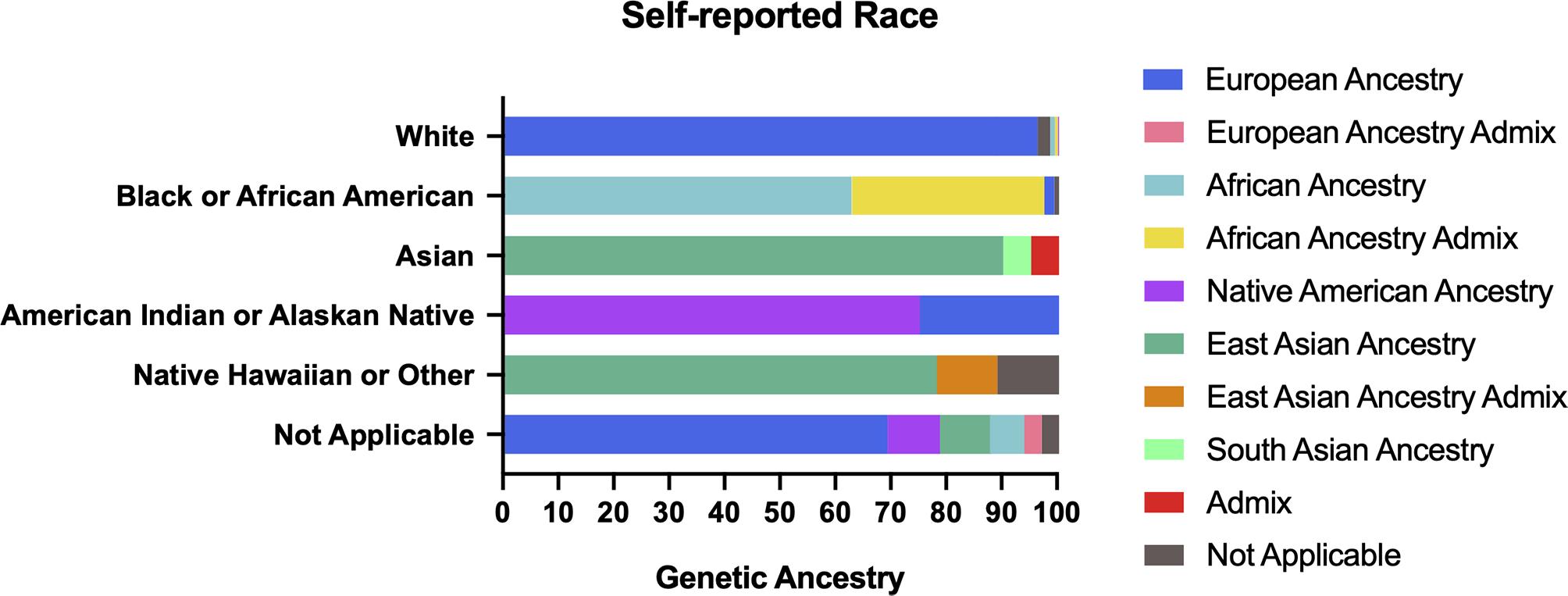

Supplement: Supplementary Figure 2 [file mmc2.jpg]
