# Supplementary material for: MECOM amplified endometrial cancer, a novel subset of copy number high tumors associated with poor prognosis
Source: Gynecol Oncol Rep. 2025 Nov 16;62:101993. doi: 10.1016/j.gore.2025.101993 (PMC12670447; doi:10.1016/j.gore.2025.101993)
Supplement: Supplementary Data 3 [file mmc3.docx]

S3. Prognostic Impact of Histology, Race and Stage on Progression-Free and Overall Survival

| Covariates | Unadjusted Hazard Ratio (95% CI) | p-value | *MECOM* amp  aHR (95% CI) | p-value | *MECOM^#^* amp  aHR (95% CI) | p-value | *CCNE1* amp  aHR (95% CI) | p-value | *ERBB2* amp  aHR (95% CI) | p-value |
| --- | --- | --- | --- | --- | --- | --- | --- | --- | --- | --- |
| Progression-Free Survival | | | | | | | | | | |
| High-grade histology  Black race  Stage III/IV | 2.3 [1.50-3.51] | 0.0001 | 1.5 [0.92-2.37] | 0.1060 | 1.6 [0.99-2.65] | 0.0526 | 1.7 [1.09-2.73] | 0.0194 | 1.7 [1.09-2.70] | 0.0194 |
|  | 1.3 [0.85-2.00] | 0.2290 | 0.9 [0.56-1.43] | 0.6445 | 1.0 [0.58-1.69] | 0.9807 | 1.0 [0.64-1.63] | 0.9277 | 1.0 [0.64-1.61] | 0.9403 |
|  | 3.5 [2.46-5.06] | <0.0001 | 2.6 [1.75-3.96] | <0.0001 | 2.9 [1.87-4.56] | <0.0001 | 2.8 [1.85-4.19] | <0.0001 | 2.8 [1.84-4.17] | <0.0001 |
| Overall Survival | | | | | | | | | | |
| High-grade histology  Black race  Stage III/IV | 3.7 [2.09-6.62] | <0.0001 | 2.7 [1.43-5.04] | 0.0021 | 2.5 [1.32-4.81] | 0.0049 | 3.2 [1.73-5.90] | 0.0002 | 3.1 [1.68-5.67] | 0.0003 |
|  | 1.2 [0.70-1.96] | 0.5442 | 0.8 [0.46-1.41] | 0.4573 | 1.1 [0.58-1.99] | 0.8259 | 1.0 [0.56-1.67] | 0.8882 | 0.9 [0.54-1.59] | 0.7805 |
|  | 4.1 [2.69-6.31] | <0.0001 | 3.0 [1.82-4.76] | <0.0001 | 2.9 [1.69-4.91] | 0.0001 | 3.1 [1.95-5.05] | <0.0001 | 3.1 [1.89-4.91] | <0.0001 |

Adjusted Hazard ratio (aHR)

^#^*MECOM* amplification without co-amplification of *CCNE1* or *ERBB2*
